# Multi-Mode Estimation for Small Fixed Wing Unmanned Aerial Vehicle Localization Based on a Linear Matrix Inequality Approach

**DOI:** 10.3390/s17040887

**Published:** 2017-04-18

**Authors:** Mostafa Elzoghby, Fu Li, Ibrahim. I. Arafa, Usman Arif

**Affiliations:** 1 School of Automation Science and Electrical Engineering, Beihang University, 37 Xueyuan Road, Haidian District, Beijing 100191, China; fuli@buaa.edu.cn (F.L.); usman.arif@buaa.edu.cn (U.A.); 2 School of Control and Automation, MTC, Al-Khalifa Al-Maamoon Street Kobry Elkobbah, Cairo 11331, Egypt; dr.ibrahim_arafa@yahoo.com

**Keywords:** integrated navigation system, multi-mode estimation, sensor data fusion, Luenberger observer, small UAV localization

## Abstract

Information fusion from multiple sensors ensures the accuracy and robustness of a navigation system, especially in the absence of global positioning system (GPS) data which gets degraded in many cases. A way to deal with multi-mode estimation for a small fixed wing unmanned aerial vehicle (UAV) localization framework is proposed, which depends on utilizing a Luenberger observer-based linear matrix inequality (LMI) approach. The proposed estimation technique relies on the interaction between multiple measurement modes and a continuous observer. The state estimation is performed in a switching environment between multiple active sensors to exploit the available information as much as possible, especially in GPS-denied environments. Luenberger observer-based projection is implemented as a continuous observer to optimize the estimation performance. The observer gain might be chosen by solving a Lyapunov equation by means of a LMI algorithm. Convergence is achieved by utilizing the linear matrix inequality (LMI), based on Lyapunov stability which keeps the dynamic estimation error bounded by selecting the observer gain matrix (L). Simulation results are presented for a small UAV fixed wing localization problem. The results obtained using the proposed approach are compared with a single mode Extended Kalman Filter (EKF). Simulation results are presented to demonstrate the viability of the proposed strategy.

## 1. Introduction

The major issue in the field of autonomous Unmanned Aerial Vehicles (UAVs) and Micro Aerial Vehicles (MAVs) is their consistent and precise localization. This problem is usually solved by combining navigation data provided by different sensors. Recently, the problem has attracted many researchers and a lot of work has been done in the autonomous low cost UAV guidance, navigation and control (GNC) area, leading to great success during the last few years. Multiple sensor information fusion for robot navigation increases the accuracy and robustness of the navigation information [1]. Different methods of integration of navigation data from different types of sensors and estimators have been developed and investigated by researchers in the recent past few years. The focus has mainly been on the integration of Inertial Navigation System (INS) information with GPS to provide precise navigation information, but GPS data is occasionally unavailable due to outages. GPS is a satellite-based navigation system that provides accurate positioning information anyplace on the globe, but GPS is insufficient for many navigation applications as a stand-alone system because while GPS has superior long-term stability and error performance, the accuracy of GPS measurements can be poor for short periods of time due to several errors related with its signal acquisition. INS, on the other hand, is not influenced by external interferences, and has higher bandwidth with good short-term accuracy, stability and noise characteristics, but it possesses poor long-term reliability as the navigation accuracy degrades with time because of bias drift and noise, and therefore it needs external information from GPS measurements for correction and initialization for long-term, high accuracy navigation. GPS/INS integrated systems are robust, operate at higher bandwidth, provide reliable navigation information and have better noise characteristics with the long-term stability of GPS and improved positioning continuity compared to either stand-alone GPS or INS [2,3,4]. Nowadays, the trend is to increase the number of navigation sensors to mitigate the GPS dependence. All sources positioning navigation (ASPN) is an important concept in a new era of navigation studies to achieve low cost, effective, and robust navigation solutions regardless of the availability of a global positioning system (GPS) [5]. Along with the importance of increasing the number of navigation information sources, the design of integrated navigation system using estimators is important to yield more accurate, robust and reliable integrated systems. The key problem of any integrated navigation system is how to achieve an optimal estimation for position, velocity, attitudes and state of interest parameters.

Multi-Sensor Data Fusion (MSDF) is defined as the acquisition and processing of data collected by different sensors. There are different MSDF techniques, ranging from Kalman filtering methods to those used in soft computing technology, while some work has also been done on hybrid architectures [6].

The need for multiple model architectures emerges in numerous applications where framework changes or alternative models are more suitable to depict a particular issue. Cases of such issues are fault diagnosis, linearization of non-linear frameworks with the end goal of control, reconfiguration, and so on. Multi-mode state estimation frameworks turn out to be more troublesome than standard single-model estimations and extensive research has focused on this matter [7].

Multiple-model estimation techniques for the most part expect that the actual model has a place within a finite arrangement of models. A priori event probability for every mode is known and what's more, the system equations and measurement equations are generally corrupted by noises. In addition, the change starting with one framework mode then onto the next one is frequently depicted by a Markov chain method. The deduction of the optimal estimator is troublesome regardless of the possibility that the noises are thought to be independently Gaussian distributed.

As a result, various approximate techniques have been developed. Most techniques depend on the residual generation executed by a bank of filters (each implemented in accord with a particular model) and a hypothesis testing algorithm which is used to processes the residuals to decide on a conditioned-model estimate of state. The residuals give an assessment of how near each of the filter models is to the actual model. Such techniques are for the most part alluded to as multiple-model (MM) estimation approaches [8]. Upgrades have been introduced by exchanging the computed statistics between the different filters of the bank [9] (this is normally designated as an Interacting Multiple Model (IMM) technique). IMM techniques are widely applied in target tracking [10]. Numerous uses of these techniques are surveyed in the literature (see, among others, [11]).

The computational complexity cost of an arrangement of parallel filters may prevent one from executing them online for a high order system with a large number of state variables, and algorithms have been proposed to diminish the amount of calculation needed. A conceivable strategy to address this disadvantage comprises choosing the number of residual generators (thus the computational cost is assigned) and after that an adaptive tuning of each filter can be introduced [12]. Another strategy is alluded to as multiple-model estimation with variable structure and depends on the usage of a variable arrangement of adaptable models [13]. Expansions to a more broad class of hybrid framework have been reported [14,15].

More formal strategies to manage a multiple-model estimation issue have been introduced as of late and provide convergence results. These estimation issues might be solved utilizing limited dimensional filters, as pointed out in [16]. Various algorithms are introduced in [17] and depend on different Bayesian cost equations. A strategy to manage non-Gaussian noises is introduced in [18].

Here the consideration is on the issue of reproducing the state of a dynamic framework based on a switching environment in general. The goal is to build up an estimator ready to track the state of the dynamic system framework based on a limited amount of data regarding the techniques cited above. More specifically, we suppose that different measurement information is available from various sensors (such as GPS, IMU, altimeters … etc.) at different time instants and in addition, the GPS signals can fade or become corrupted in many availability over time cases and therefore they are degraded. Furthermore, we assume no noise influences in the measurements and dynamic equations and thus any uncertainty is just due the initial state and switching between modes. Such presumptions are relaxed in the simulations to get a more practical evaluation comparison, where the system noise and measurement noise have been introduced. The switching model is not considered and the probability information is not given. Thus, a decision maker depends on the evaluation of the residual generation that is performed based on the difference between the measured system output for each possible operating mode and the estimated system output from the continuous observer. The assessment of the residuals applies a reset to the filter model according to the matching operating mode. As an outcome, the dynamics error of the eventual scheme acts as a switching system. The problem in any kind of switching observer among different modes is the observer gain selection to guarantee the stability of the estimation dynamic errors and this problem remains difficult to solve and harder than the design of the standard observer. The observer gain in case of the classical Luenberger observer is chosen on the basis of the pole placement technique where convergence of the error dynamics might occur only if the poles of the dynamics error are placed in the strictly stable region, while in case of the observer in a switching environment then the solution is by seeking the common Lyapunov function. The stability is guaranteed for such an error dynamics by the solution of a common Lyapunov function in the ideal case when the system is not influenced by the noise and the right decision for switching is taken at every time instant [19]. Linear matrix inequality (LMI) techniques are appropriate to discover this solution and also to permit a more effective design of the observers to improve the estimation performance [20]. In addition, an enhancement has been made by executing a projection strategy [21,22] on the Luenberger observer by incorporating the last measurements into the estimation process to update the current estimate. Such an enhancement results in higher estimation performance by reducing the estimation error. Some of current research works focus on observer-based controller design scheme via using the relation of Young inequality in a judicious manner to calculate the observer and controller gains based on improved LMI conditions to ensure the stability of the closed-loop system [23,24,25].

In our simulation we have selected the Extended Kalman Filter (EKF) to compare with the proposed approach because EKF is a successful and widespread estimator for nonlinear systems and is used in many applications [26] and it is well proven for inertial data fusion [27]. The Extended Kalman Filter is widely used for the purpose of sensor fusion in UAV applications, In [28,29,30,31] robust navigation for fixed wing Unmanned Aerial Systems (UASs) based on integration of MSDF architecture using an Extended Kalman Filter has been introduced. Another integrated navigation system for UAV localization using the method of nonlinear smooth variable structure filter is presented and compared with an Extended Kalman Filter in [32]. Moreover, in [27,33] the authors evaluated the Extended Kalman Filter for inertial data fusion-based navigation localization for mobile robots. EKF has been most widely used for the application of multi-sensor fusion, either in a self-sufficient, or in a multiple model manner. The main reason behind the widespread use of EKF is the lower computational cost and simplicity in dealing with nonlinear systems. Many researchers have modified the multiple model approach using EKF as per the system design and requirements. In [34] a multiple model approach using EKF is described for collision avoidance and position estimation, and a similar approach for multi-target state estimation is presented in [35].

The article organization is as follows: multi-mode estimation based on a switching observer has been described in Section 2, Then feasible solutions for the stability and performance are discussed in Section 3 and Section 4, where the stability analysis of the estimation error has been addressed in Section 3 while the performance enhancement-based projection consideration has been addressed in Section 4. Finally, in Section 5, the simulation framework and results, discussion and a comparison are given to show the effect of the proposed method.

## 2. Multi-Mode Estimation-Based Switching Observer

Let a discrete time linear system be described by:

(1) $$x\left(k+1\right)=A_{\sigma \left(k\right)}\;x\left(k\right)+B_{\sigma \left(k\right)}\;u\left(k\right)+w_{k},\;y\left(k\right)=C_{\sigma \left(k\right)}\;x\left(k\right)+v_{k}$$

where x(k) is the state vector, u(k) is the input vector, y(k) is the measurement vector,$\;w_{k}\;$is process noise, $v_{k}\;$is measurement noise and σ(k) is a function of switching signal which maps between the index of the current time step among a set of indices {1,2,.., k}. Every index from these indices is compatible with various system and measurement equation configuration models, i.e., {$A_{\sigma \left(k\right)},\;B_{\sigma \left(k\right)},\;C_{\sigma \left(k\right)}$} ∊ {($A_{1},\;B_{1},\;C_{1}$), ($A_{2},\;B_{2},\;C_{2}$)... ($A_{k},\;B_{k},\;C_{k}$)}. We suppose the pairs ($A_{i},\;C_{i}$), i=1, 2,.., k are observable, and also presume that the switching sequence is arbitrary and no a priori information on the switching probability from one mode into another one is required. A switching Luenberger observer for (1) is as follows:

(2) $$\hat{x}\left(k+1\right)=A_{\sigma \left(k\right)}\;\hat{x}\left(k\right)+B_{\sigma \left(k\right)}\;u\left(k\right)+L_{\sigma \left(k\right)}\;r_{\sigma \left(k\right)},\;k=1,2,\ldots$$

where $L_{\sigma \left(k\right)}$ and $r_{\sigma \left(k\right)}$ are the observer gain matrix and the residual at the time *k*, respectively.

We suppose that the different measurement information is available from various sensors at different discrete time instants and in addition, some of these sensors may be not available at different time instants, such as the GPS signals that can fade or become corrupted in many cases therefore degrading their availability and as a consequence, we assume that the measurement information from different sensors can be grouped into a multi-measurement mode to ensure the information availability from different sensors in the absence of GPS information to keep describing the system dynamics all the time. This stage acquires the measurements at every time step and estimates the system mode based on the evaluation of the residuals. The system output is then generated by this most likely system mode. The proposed hybrid navigation system combines all the measurements from all the sensors through two operational modes, designated as mode 1 and 2.

An essential question is the assessment of the residuals, i.e., how to decide a choice on the working operating mode utilizing the residuals. As the error dynamics are supposed to be stable, a possible assessment technique comprises choosing the mode that relates to the most reduced value of the residuals. For each system mode, we calculate the residual of the measurement with the output of the corresponding mode. The residual is then used to decide the operating mode; such a decision relies on the criterion used to discriminate among the different residuals, the resulting design scheme comes out to behave like a switching system. A general debate on switching system frameworks is beyond the scope of this article, and for an introduction readers can consult [19]. It is worth mentioning here that we don’t depend on a probability depiction of stochastic variables and events in general, and then a traditional Bayesian way to deal with this issue is prevented. In this context, a feasible solution comes out to be that of choosing the model with the smallest matching residuals.

Generally, residuals are generated from the difference between the measured system output y(k) for each mode and the estimated system output$\;\hat{y}\left(k\right)$:

(3) $$r_{i}(k)y_{i}(k)-{\hat{y}}_{i}\left(k\right)=y_{i}\left(k\right)-C_{i}\hat{x}(k),i=1,\;2,...,\;N$$

**Figure 1:**
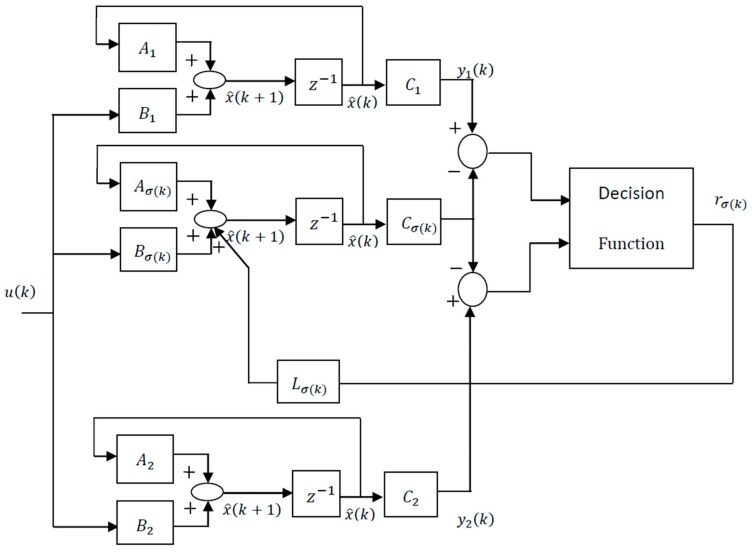
Scheme of the multi-measurement mode estimation-based switching observer.

The switching between the two modes, depending on the decision function can be expressed as:

(4) $$\mathrm{decision}\;\mathrm{function}=\{\begin{array}{cc} if\;RMS\|r_{1}\left(t\right)\|\leq \epsilon \;and\;t=t_{1} & switch\;to\;mode\;one\\ otherwise\;RMS\|r_{2}\left(t\right)\|\leq \epsilon \;or\;t\neq t_{1} & switch\;to\;mode\;two \end{array}$$

where ϵ is a root mean square of the residual threshold and $t_{1}$ is the instant at which all measurements are available including the GPS. The decision function not only judges the availability of GPS, but also judges the accuracy of the sensors’ measurements where the switching between the two modes is based on the most reduced residual which leads to the most likely mode. This technique acquires the measurements at every time step and estimates the system mode. As a consequence, the switching observer can be described by the following expression:

(5) $$\hat{x}\left(k+1\right)=A_{i}\;\hat{x}\left(k\right)+B_{i}\;u\left(k\right)+L_{i}\;\left(y_{i}\left(k\right)-\;C_{i}\hat{x}\left(k\right)\right),\;i=1,2,\ldots .,$$

where, $\hat{x}\left(0\right)=x\left(0\right)$ is chosen a priori, $y_{i}\left(k\right)$ is the measurement vector of the ith mode and $L_{i}$ is the related observer gain. The multi-measurement mode estimation-based switching observer scheme is depicted in Figure 1.

The essential problems emerging in the design of such an observer are related to stability, optimization, and performance. These problems will be addressed in the next sections and possible solutions will be proposed.

## 3. Stability Analysis of the Estimation Error

The attention in the first place is placed on the stability of the estimation dynamics error $e\left(k\right)=\;x\left(k\right)-\hat{x}\left(k\right)$. Such error dynamics behave as a switching dynamic system, consequently the stability is guaranteed for such an error dynamics case by the solution of a common Lyapunov function [36]. By taking into consideration the system in Equation (1) and with the assumption that the pairs (*A_i_*, *C_i_*) are observable, and if matrix P exists as a solution of the following Lyapunov inequality:

(6) $${\left(A_{i}-L_{i}C_{i}\right)}^{T}P\left(A_{i}-L_{i}C_{i}\right)-P<0\;,\;i=1,2,\ldots N$$

where, matrix P is a symmetric positive definite then the observer in Equation (2) includes a dynamic estimation error that converges exponentially asymptotically to zero [36]. To prove this result then, we recall the error dynamics equation as follows:

(7) $$e\left(k+1\right)=\;x\left(k+1\right)-\hat{x}\left(k+1\right)$$

From the system Equation (1) and from the observer Equation (2) we can substitute in the error dynamic Equation (7) then:

(8) $$e\left(k+1\right)=A_{\sigma \left(k\right)}\;x\left(k\right)+B_{\sigma \left(k\right)}\;u\left(k\right)-A_{\sigma \left(k\right)}\;\hat{x}\left(k\right)-B_{\sigma \left(k\right)}\;u\left(k\right)-L_{\sigma \left(k\right)}\;r_{\sigma \left(k\right)}$$

where, the residual $r_{\sigma \left(k\right)}=\;y_{\sigma \left(k\right)}\left(k\right)-\;C_{\sigma \left(k\right)}\hat{x}\left(k\right)$ then:

(9) $$e\left(k+1\right)=A_{\sigma \left(k\right)}\;x\left(k\right)-A_{\sigma \left(k\right)}\;\hat{x}\left(k\right)-L_{\sigma \left(k\right)}\left(y_{\sigma \left(k\right)}\left(k\right)-\;C_{\sigma \left(k\right)}\hat{x}\left(k\right)\right)\;e\left(k+1\right)=A_{\sigma \left(k\right)}\left(\;x\left(k\right)-\;\hat{x}\left(k\right)\right)-L_{\sigma \left(k\right)}\left(C_{\sigma \left(k\right)}\;x\left(k\right)-\;C_{\sigma \left(k\right)}\hat{x}\left(k\right)\right)\;e\left(k+1\right)=A_{\sigma \left(k\right)}\left(\;x\left(k\right)-\;\hat{x}\left(k\right)\right)-L_{\sigma \left(k\right)}C_{\sigma \left(k\right)}\left(\;x\left(k\right)-\;\hat{x}\left(k\right)\right)\;e\left(k+1\right)=\left(A_{\sigma \left(k\right)}-L_{\sigma \left(k\right)}C_{\sigma \left(k\right)}\right)\left(\;x\left(k\right)-\;\hat{x}\left(k\right)\right)\;e\left(k+1\right)=\left(A_{\sigma \left(k\right)}-L_{\sigma \left(k\right)}C_{\sigma \left(k\right)}\right)e\left(k\right)$$

With the consideration of the Lyapunov function is $V_{k}=\;e_{k}^{T}Pe_{k}$, we get $V_{k+1}\leq \;V_{k}$, if:

(10) $${\left(A_{\sigma \left(k\right)}-L_{\sigma \left(k\right)}C_{\sigma \left(k\right)}\right)}^{T}P\left(A_{\sigma \left(k\right)}-L_{\sigma \left(k\right)}C_{\sigma \left(k\right)}\right)-P<0\;,$$

where P is a positive symmetric definite matrix, then the Equation (6) can be derived easily.

The assumption of $\left(A_{i},\;C_{i}\right)$ are observable is an important feature to ensure the inequality in Equation (6) allows a solution of a symmetric positive matrix P, however the presence of such a matrix P is required for achieving the inequalities which guarantee the stability. Therefore, the goal is to get the solution of the following observer gain $L_{i}$ problem, such that a matrix P exists for the solution of Lyapunov inequalities:

(11) $${\left(A_{i}-L_{i}C_{i}\right)}^{T}P\left(A_{i}-L_{i}C_{i}\right)-P<0\;,\;i=1,2,\ldots N$$

However, the above problem is difficult to solve in this form, so, it is appropriate to choose the matrices $L_{i}$ and P simultaneously. Therefore this problem can be changed to another simpler form based on the relation of the Schur complement (for proof the reader is referred to [36]), thus the Lyapunov inequality in the aforementioned problem is equivalent to [20]:

(12) $$\left(\begin{array}{cc} P & \left(PA_{i}-\;Y_{i}C_{i}\right)\\ {\left(PA_{i}-\;Y_{i}C_{i}\right)}^{T} & P \end{array}\right)>0\;,\;i=1,2,\ldots N$$

Now, we can solve the Lyapunov inequality in this form by means of LMI techniques. LMI techniques permit one to solve such problems using convex programming [20,37]. In order to efficiently perform such an optimization by linear matrix inequalities, we have used the CVX 3 toolbox as a convex programming algorithm to find the matrices P and $Y_{i}$ and then find the observer gain $L_{i}$ as follows:

(13) $$L_{i}=P^{-1}Y_{i},\;i=1,2,\ldots N$$

## 4. Performance Enhancement by Using Luenberger Observer Based Projection

**Figure 2:**
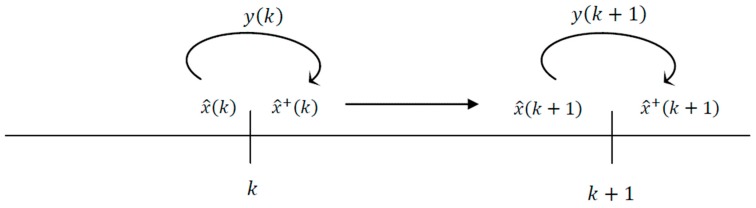
State estimation based on the last measurement.

The classic Luenberger observer in Equation (2) estimates the state vector $\hat{x}\left(k+1\right)$ at time (k+1) by using the available measurements y(k) at time k. In this section we aim to discuss an approach to compute the estimate state vector $\hat{x}\left(k+1\right)$ at time (k+1) by using the available measurement vector y(k+1) at time (k+1) just like a standard Kalman filter. We denote ${\hat{x}}^{+}\left(k\right)$ as the state estimates at time k incorporating the output at time k as shown in Figure 2. Such an approach is based on the projection method and takes into consideration the last measurement to enhance the estimation performance (for further information about such an approach the reader is referred to [36]). More specifically, this estimation technique can be characterized as follows [36,38]:

(14) $$\hat{x}\left(k+1\right)=A_{\sigma \left(k\right)}\;{\hat{x}}^{+}\left(k\right)+B_{\sigma \left(k\right)}\;u\left(k\right)+L_{\sigma \left(k\right)}\;\left(y\left(k\right)-\;C_{\sigma \left(k\right)}\;{\hat{x}}^{+}\left(k\right)\right)$$

(15) $${\hat{x}}^{+}\left(k+1\right)=\hat{x}\left(k+1\right)+P^{-1}C_{\sigma \left(k+1\right)}^{T}\;{\left(C_{\sigma \left(k+1\right)}P^{-1}C_{\sigma \left(k+1\right)}^{T}\right)}^{-1}\;\left(y\left(k+1\right)-\;C_{\sigma \left(k+1\right)}\;\hat{x}\left(k+1\right)\right)$$

where ${\hat{x}}^{+}\left(0\right)=x\left(0\right)$ is chosen a priori, $L_{\sigma \left(k\right)}$ and P are the observer gain and a symmetric positive definite matrix at time k, respectively.

The estimation error before incorporating y(k+1) is as follows:

(16) $$e\left(k+1\right)=\;x\left(k+1\right)-\hat{x}\left(k+1\right)=\left(A_{\sigma \left(k\right)}-L_{\sigma \left(k\right)}C_{\sigma \left(k\right)}\right)e^{+}\left(k\right)$$

where $e=x-\hat{x}$ and $e^{+}=x-{\hat{x}}^{+}$, then the estimation error after incorporating y(k+1) is as follows:

(17) $$e^{+}\left(k+1\right)=x\left(k+1\right)-{\hat{x}}^{+}\left(k+1\right)=\left[I-P^{-1}C_{\sigma \left(k+1\right)}^{T}\;{\left(C_{\sigma \left(k+1\right)}P^{-1}C_{\sigma \left(k+1\right)}^{T}\right)}^{-1}C_{\sigma \left(k+1\right)}\right]e\left(k+1\right)$$

Considering the Lyapunov functions are $V_{k}=\;e_{k}^{T}Pe_{k}$ and $V_{k}^{+}=e_{k}^{+T}Pe_{k}^{+}$, for stability we want to show that $e^{+}\left(k\right)$ converges to zero asymptotically by proving $V_{k}^{+}$ is decreasing in k. Then, it is sufficient to show that $V_{k+1}^{+}<\;V_{k+1}$, since it is obvious from Equation (16) that $V_{k+1}<\;V_{k}^{+}$ if the Lyapunov inequalities are satisfied. Let’s consider:

(18) $$\begin{array}{c} \;V_{k+1}^{+}=e_{k+1}^{+T}Pe_{k+1}^{+}\\ \;=\;{\left[e\left(k+1\right)-P^{-1}C_{\sigma \left(k+1\right)}^{T}\;{\left(C_{\sigma \left(k+1\right)}P^{-1}C_{\sigma \left(k+1\right)}^{T}\right)}^{-1}C_{\sigma \left(k+1\right)}e\left(k+1\right)\right]}^{T}P[e\left(k+1\right)\\ \;-P^{-1}C_{\sigma \left(k+1\right)}^{T}\;{\left(C_{\sigma \left(k+1\right)}P^{-1}C_{\sigma \left(k+1\right)}^{T}\right)}^{-1}C_{\sigma \left(k+1\right)}e\left(k+1\right)]\;\\ \;=\;V_{k+1}-\;e^{T}\left(k+1\right)\;C_{\sigma \left(k+1\right)}^{T}{\left(C_{\sigma \left(k+1\right)}P^{-1}C_{\sigma \left(k+1\right)}^{T}\right)}^{-1}C_{\sigma \left(k+1\right)}e\left(k+1\right)\;\\ \;<\;V_{k+1} \end{array}$$

In the next section, the improvements of the estimation performance which were introduced via the Luenberger observer-based projection consideration can appear clearly in the simulation results.

## 5. Simulation Framework and Results

In this section we present our simulation framework and results in order to validate the proposed multi-mode estimation by using a Luenberger observer after a projection (LOAP)-based LMI approach for the small fixed wing unmanned aerial vehicle localization problem.

The nonlinear state dynamics and measurement model which describe the dynamics of the body of the small fixed wing unmanned aerial vehicle can be expressed by the following equations:

(19) $$\dot{x}\left(t\right)=f\left(x\left(t\right),u\left(t\right),t\right),\;y\left(t\right)=h\left(x\left(t\right),u\left(t\right),t\right)$$

where x is the state vector, which consists of attitude in terms of Euler angles, position and velocity expressed in navigation frame, gyros and accelerometers biases, respectively, defined as follows:

(20) $$x=\left[\phi \;\theta \;\psi \;P_{n}\;P_{e}\;h\;V_{n}\;V_{e}\;V_{d}\;b_{\omega_{x}}\;b_{\omega_{y}}\;b_{\omega_{z}}\;b_{a_{x}}\;b_{a_{y}}\;b_{a_{z}}\;\right]$$

Furthermore, u is the system input which in our case is the IMU measurements, expressed as follows:

(21) $$u=\left[\;\omega_{x}\;\omega_{y}\;\omega_{z}\;f_{x}\;f_{y}\;f_{z}\;\right],$$

Nonlinear navigation state model can be written as:

(22) $$f(x,u)=[\begin{array}{c} \left[\begin{array}{ccc} 1 & \sin\phi \tan\theta & \cos\phi \tan\theta \\ 0 & \cos\theta & -\sin\phi \\ 0 & \sin\phi \sec\theta & \cos\phi \sec\theta \end{array}\right]\left[\begin{array}{c} \omega_{x,gyro}-b_{\omega_{x}}\\ \omega_{y,gyro}-b_{\omega_{y}}\\ \omega_{z,gyro}-b_{\omega_{z}} \end{array}\right]\\ V_{n}\\ V_{e}\\ -V_{d}\\ C_{b}^{n}\left[\begin{array}{c} f_{x,accel}-b_{a_{x}}\\ f_{y,accel}-b_{a_{y}}\\ f_{z,accel}-b_{a_{z}} \end{array}\right]\\ 0\\ 0\\ 0\\ 0\\ 0\\ 0 \end{array}]$$

where $C_{b}^{n}$ is the transformation matrix which represents a rotation from body to navigation coordinates and because it is an orthonormal matrix then one from its characteristics is $C_{b}^{n}={\left[C_{n}^{b}\right]}^{T}$ where $C_{n}^{b}$ can be characterized as:

(23) $$C_{n}^{b}=[\begin{array}{ccc} \cos\left(\theta \right)\cos\left(\psi \right) & \cos\left(\theta \right)\sin\left(\psi \right) & -\sin\left(\theta \right)\\ \sin\left(\phi \right)\sin\left(\theta \right)\cos\left(\psi \right)-\sin\left(\psi \right)\cos\left(\phi \right) & \sin\left(\phi \right)\sin\left(\theta \right)\sin\left(\psi \right)+\cos\left(\psi \right)\cos\left(\phi \right) & \sin\left(\phi \right)\cos\left(\theta \right)\\ \sin\left(\theta \right)\cos\left(\phi \right)\cos\left(\psi \right)+\sin\left(\psi \right)\sin\left(\phi \right) & \sin\left(\psi \right)\sin\left(\theta \right)\cos\left(\phi \right)-\cos\left(\psi \right)\sin\left(\phi \right) & \cos\left(\phi \right)\cos\left(\theta \right) \end{array}]$$

The basic frame for the inertial sensors used in the simulation is the body-fixed coordinate frame (body *x*–*y*–*z* coordinate) where, *x*-axis point in the forward direction, *y*-axis point to right, and *z*-axis points downwards however, the local geodetic coordinates are North-East-Down (NED).

**Figure 3:**
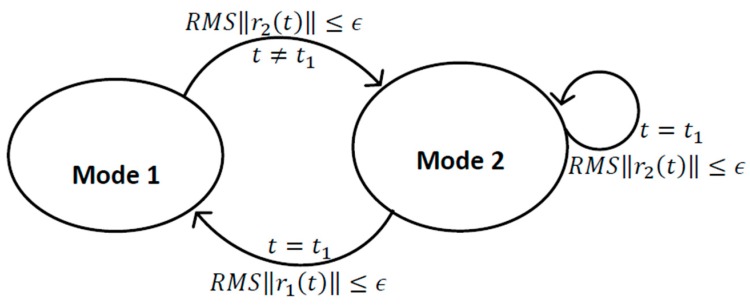
Graphical representation for switching between two modes.

In the simulation we assume that various measurement information data is available from different sensors at various different time instants and furthermore, some of these sensors might be not available at various moments of time, for example, the GPS signals can fade or be corrupted in many situations consequently degrading the availability and as an outcome of this we suppose that the measurement information data from various sensors is to be gathered into two-modes to guarantee the availability of the measurement data from various sensors in the absence of the information from some of them to keep describing the system dynamics all the time. Figure 3 shows a graphical representation for switching between two modes.

In mode one, the observations for velocity and position are obtained from GPS, while in mode two, the position and velocity in the north and east directions are acquired from the IMU and the height from a barometric pressure sensor and finally the down velocity from the fusion between barometer and vertical accelerometer measurements. The vertical velocity can be gathered on the basis on the available vertical acceleration and altitude measurements [39].

The estimation of the continuous states is performed by utilizing a Luenberger observer based on geometrical considerations, as portrayed in Section 4, and no a priori statistical information data about system and measurement noises and system uncertainties is required. Furthermore, we assume no noise influences on the measurement and dynamic equations, thus any uncertainty is just because of the initial state and switching between modes. Such presumptions are relaxed in the simulations to get a fair evaluation comparison, where the system noise and measurement noise have been introduced. The initial states are chosen a priori while the system process covariance matrix P and the observer gain matrix L are selected simultaneously by solving the Lyapunov inequality by means of the LMI approach. No a priori information about the process covariance matrix P is required, but the matrix P as a positive symmetric definite matrix is chosen as a solution for the Lyapunov inequality by means of the LMI technique. The existence of the positive definite matrix *P* as a solution of the Lyapunov inequality ensures the dynamic estimation error converges exponentially to zero. For simple denotation we call the proposed continuous observer a Luenberger observer after projection (LOAP). We tested the proposed projection filter on a class of nonlinear system and compared the results with the Extended Kalman Filter (EKF). The initialization of the state estimate for both the LOAP and EKF is the same and based on utilizing the first measurement of GPS to provide an initial position and velocity state estimate as follows 0.31 m, 2.23 m and 160.6 m for the initial north, east and height positions state estimate, respectively, and 20.8 m/s, 5.9 m/s and −1.1 m/s for the initial north, east and down velocities state estimate, while the initialization of the other state estimates such as the Euler angles, gyros biases and accelerometers biases have been set to zero. Although we initialized the position and velocity state estimate for the EKF, we assume the EKF filter started with large initial uncertainties compared to the expected magnitudes of the states to build the initial process covariance matrix P with deviations equal to 0.5 rad, 100 m and 10 m/s for the initial attitudes, position and velocity uncertainties, respectively, also we use a priori information about the initial uncertainties in the gyro and accelerometer biases equal to 0.01 rad/s and 0.1 $m/s^{2},$ respectively. Also, we design the process noise covariance matrix for EKF based on how much we expect the deviation of the reality from the model of the vehicle motion to be. Although no a priori statistical information is required for the LOAP, we applied system process noise for the LOAP the same as EKF with deviations equal to 0.002 rad, 0.5 m, 0.2 m/s for the attitude, position and velocity process noises, respectively, and assigned the process noises of the gyro and accelerometer sensors biases near to zero because we do not expect them to vary very quickly with deviations equal to $10^{-6}$ rad/s and $10^{-6}\;m/s^{2}$ for the gyro and accelerometer biases process noise, respectively. Table 1 shows the type of the measured data and the sampling rate used for each sensor while Table 2 shows a comparison of the factors of the filters used in this study.

**Figure 4:**
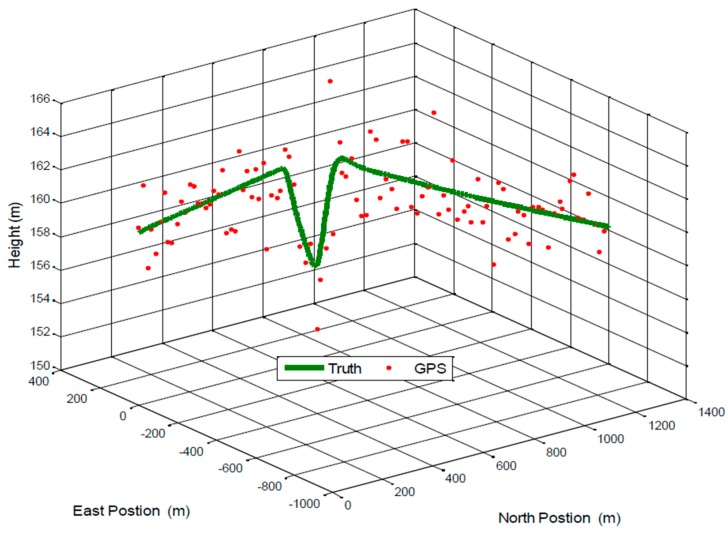
3D position trajectory of a small UAV.

A trajectory of 100 s is used in the simulation of the aforementioned model. Figure 4 provides the 3D position trajectory for the true positions and GPS measurement.

The results of our proposed approach are compared with single mode Extended Kalman Filter technique and the simulation is divided into two sections, the first is the case where the GPS signal is available at every 1 Hz, while the second is a case where the GPS signal is absent during an interval of time. The data is recorded and processed offline.

### 5.1. Evaluation of Estimation Performance (in the Case of a GPS Signal Available Every 1 Hz)

**Figure 5:**
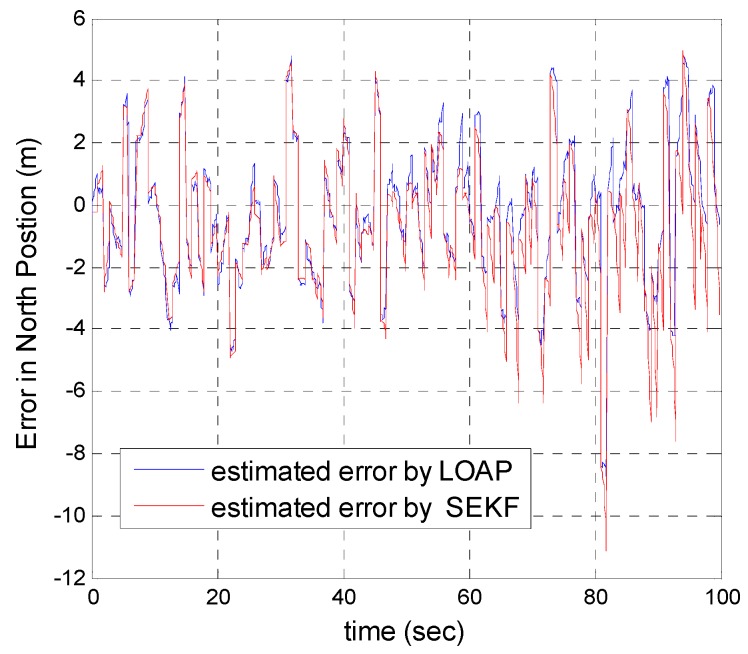
Error estimation of north position.

**Figure 6:**
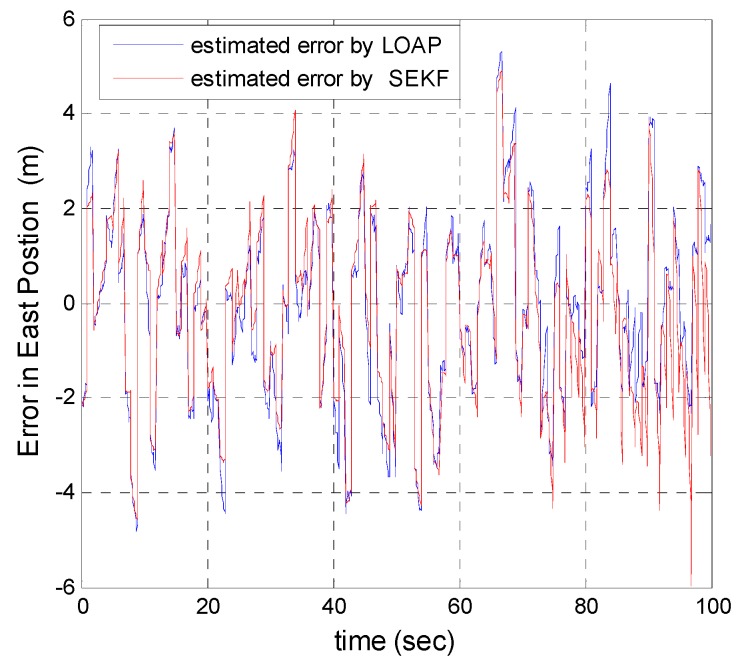
Error estimation of east position.

**Figure 7:**
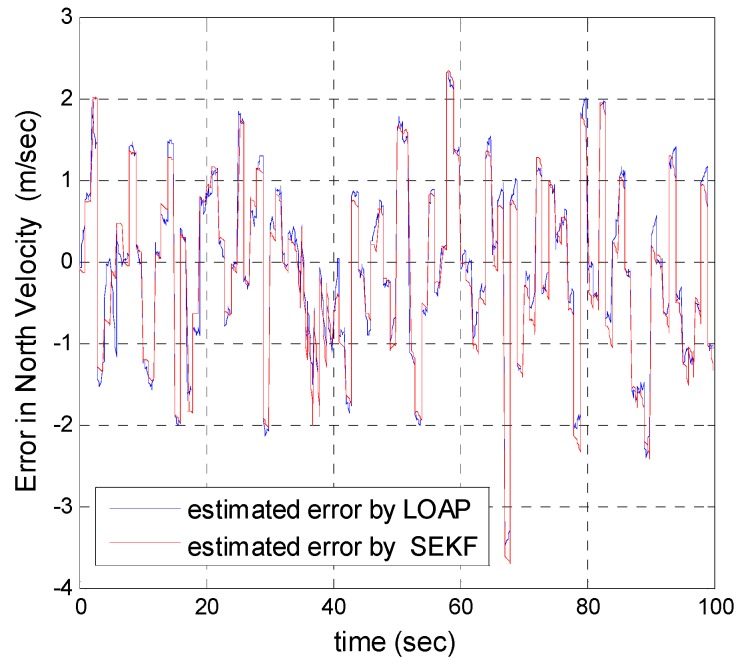
Error estimation of north velocity.

**Figure 8:**
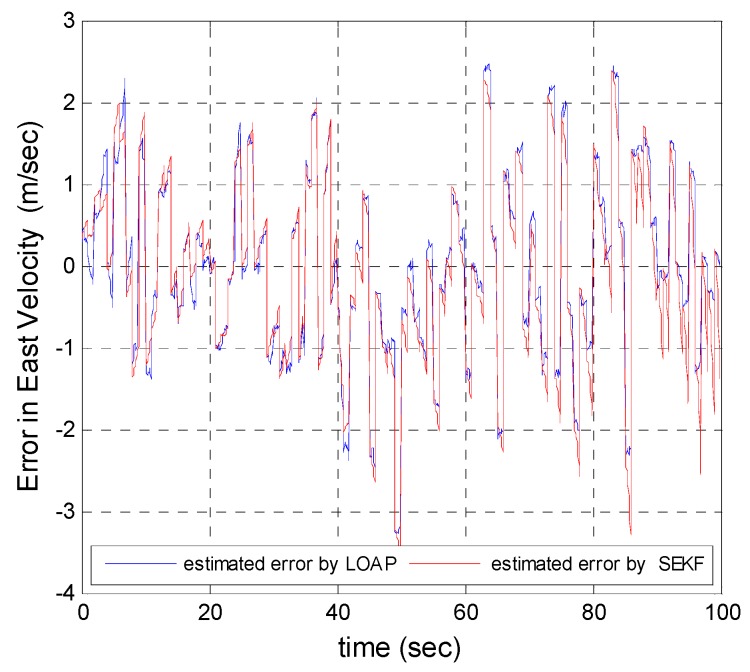
Error estimation of east velocity.

**Figure 9:**
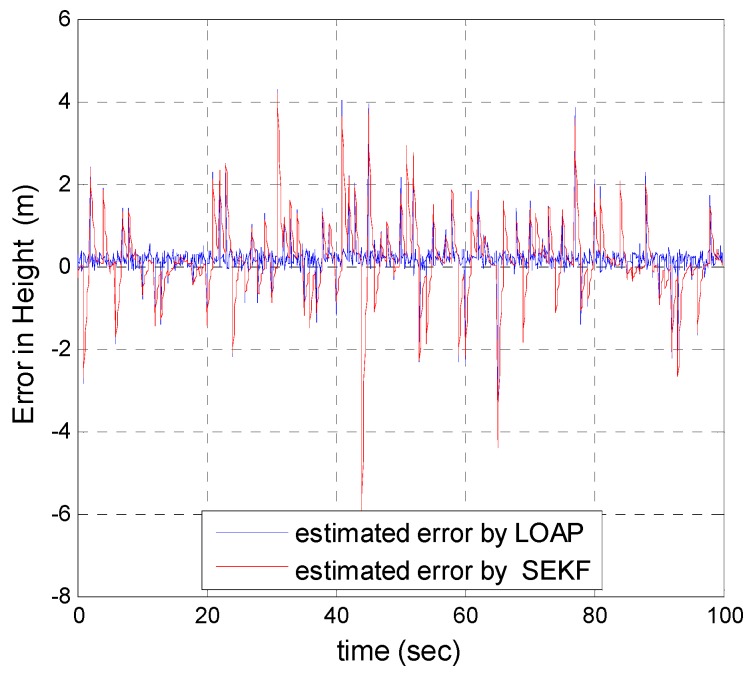
Error estimation of height.

**Figure 10:**
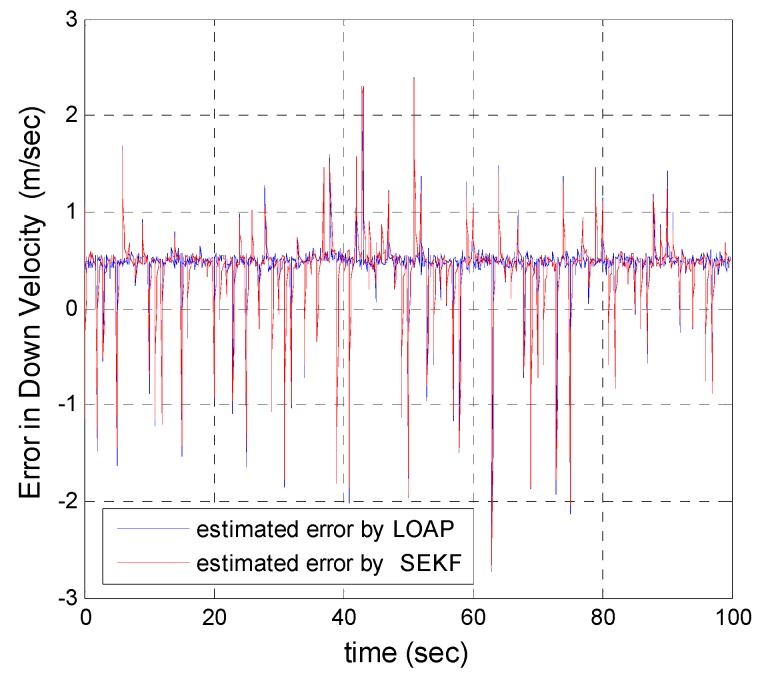
Error estimation of down velocity.

A trajectory of 100 s is used in the simulation. We use the same measurement sources for both multi-measurement mode estimation-based LOAP and single mode EKF and suppose the GPS signal is available every 1 Hz for both of them. For the latter estimator, when the GPS signal is available we assign the sampled measurement uncertainty matrix where the GPS has a measurement error with deviations equal to 2 m in position and 1 m/s in velocity, while the former does not require a priori statistical information about the measurement error, but the randomness of the sensor errors has been considered in the simulation. As the sensor error is random and every sensor has its own measuring error, the simulation has done based on extensive Monte Carlo runs. Also, for EKF at every 10 Hz when the GPS is unavailable and since the measurement depends on the other sensors we increase the values of the measurement errors in position and velocity by multiplying by the same deviations when the GPS is available by scale factor 10 and that is because the measurement position and velocity from the GPS is more accurate than the measurement position and velocity from the IMU-based MEMS but this increases the measurement noise just applied on the position and velocity in the east and north directions without the height and down velocity because of the use of a barometric altimeter to measure the height in case the GPS is unavailable with measurement noise deviation equal to 1 m. It is obvious from the Figure 5, Figure 6, Figure 7, Figure 8, Figure 9 and Figure 10 that when the GPS signal is available without any disconnection every 1 s during the time of flight then the proposed multi-measurement mode-based LOAP and the single mode EKF almost perform equally, with slightly differences between both of them where the deviations of the estimated position errors in Figure 5, Figure 6 and Figure 9 and the deviations of the estimated velocity errors in Figure 7, Figure 8 and Figure 10 are as summarized in Table 3.

Table 3 presents and compares the standard deviation of the estimated position and velocity errors in the north, east and down directions between the multi-measurement mode-based LOAP and single mode EKF. It is clear from this table that the proposed approach provides a slightly more accurate position and velocity estimation than single mode EKF. Also, the simulation results prove that the proposed approach is well suited to deal with nonlinear systems. The performance improvement of the proposed multi-measurement mode-based LOAP approach compared to the single mode EKF approach can appear clearly when the signal of GPS is fading and not available at every 1 Hz intervals. This is the object of the next simulation.

### 5.2. Evaluation of Estimation Performance (in Case of GPS Signals Not Available during an Interval Period)

**Figure 11:**
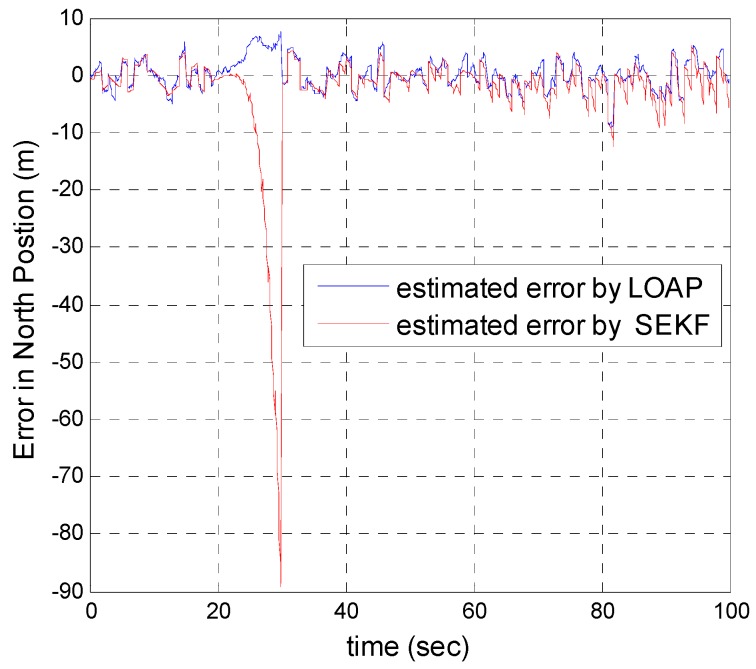
Error estimation of north position.

**Figure 12:**
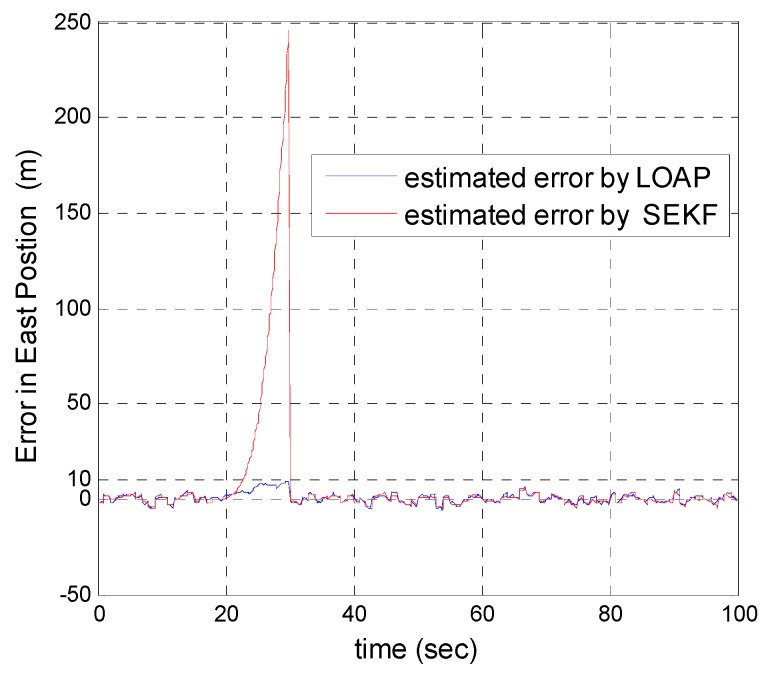
Error estimation of east position.

**Figure 13:**
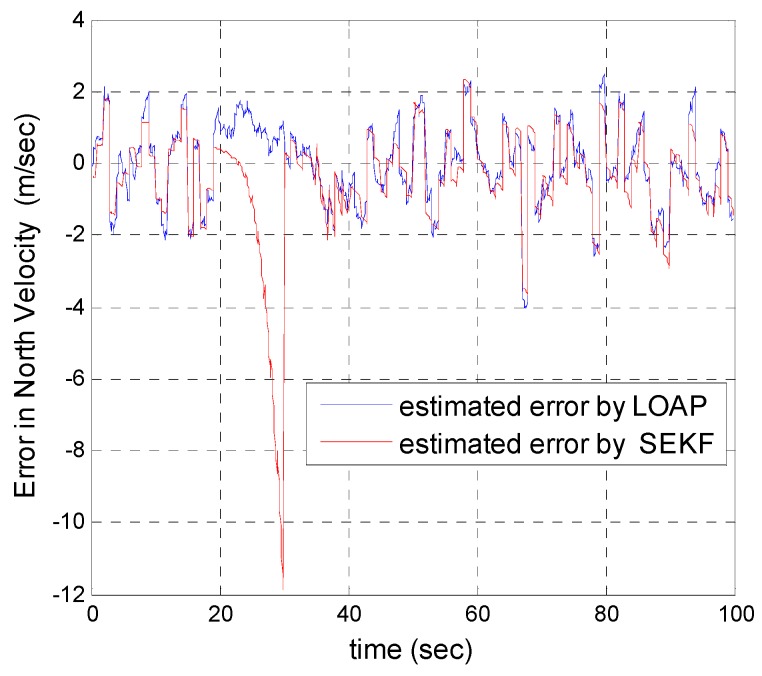
Error estimation of north velocity.

**Figure 14:**
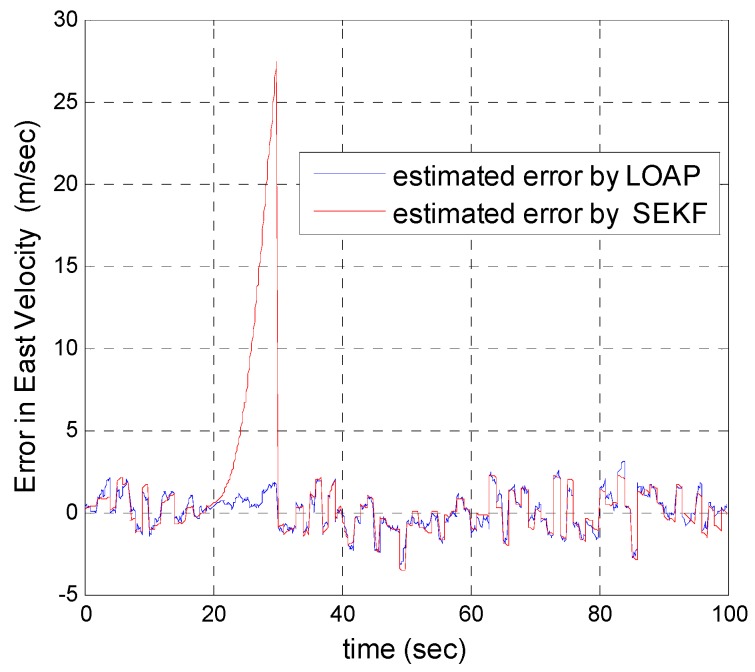
Error estimation of east velocity.

In our second simulation, we suppose that the GPS signal is disconnected and not available during the time from 20 s until 30 s. All the previous aforementioned simulation modeling conditions are the same for both the multi-measurement mode-based LOAP and the single mode EKF, except in case of single mode EKF we decreased the measurement errors for position and velocity in the east and north directions during the interval time when the GPS is disconnected to place more trust in the other sensors in the absence of the GPS signals, while the measurement error of height and down velocity remain the same as in the previous simulation because we are using a barometric altimeter to provide the height. From the simulation results in Figure 11 and Figure 12 we notice that the estimation error of the north and east positions in the case of the single mode EKF grows exponentially and increases quickly, reaching 89.355 m and 245.4513 m, respectively, while in the case of the multi-measurement mode-based LOAP the estimated position error remains stable and bounded within the acceptable range, increasing slowly and reaching 7.8117 m and 9.715 m for the north and east, respectively. Also, it is obvious from Figure 13 and Figure 14 with respect to the estimation error of the north and east velocities in case of the single mode EKF, it grows exponentially, increasing quickly and reaching 11.88 m/s and 27.4918 m/s, respectively; while in the case of the multi-measurement mode the estimated velocity error remains stable within less than 1.7622 m/s for the north and reaching 1.8424 m/s for the east direction.

**Figure 15:**
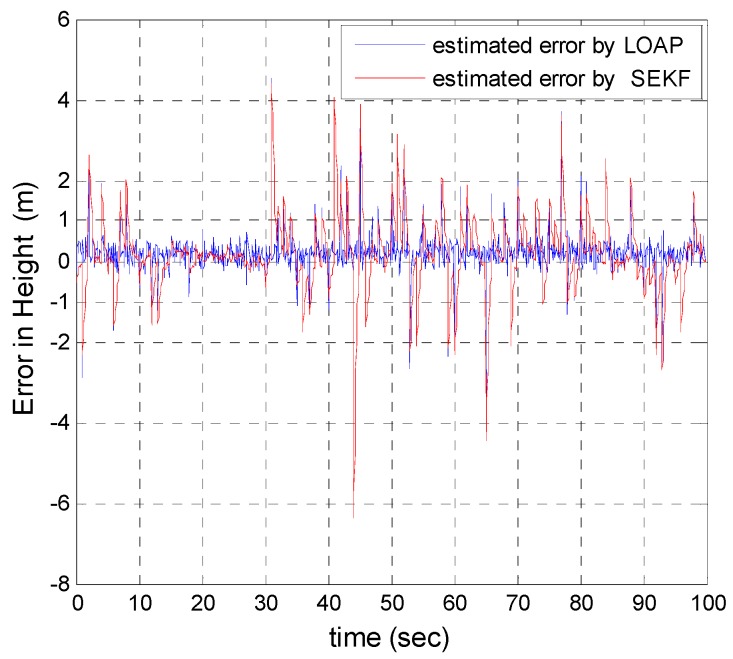
Error estimation of height.

**Figure 16:**
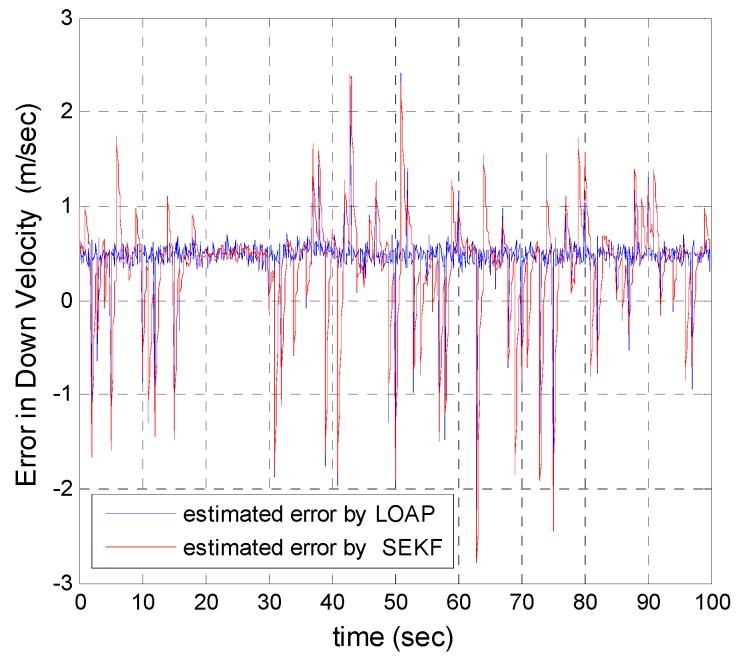
Error estimation of down velocity.

As a result, the superiority in the case of multi-measurement mode-based LOAP over the single mode EKF is obvious because when the GPS is not available at the instant of time which all the measurements should be available, the switching observer-based LOAP makes the right decision to switch to the second mode to guarantee the availability of the position and velocity measurements and then the stability of the estimation errors is obtained by solving the Lyapunov inequality-based stability by means of the LMI approach and this keeps the dynamic estimation error bounded by selecting the observer gain matrix (L). However, as shown in Figure 15, the estimation accuracies in the vertical channel have been improved in the absence of GPS because of the height measurement acquired every 10 Hz from a barometric altimeter with higher accuracy than GPS in the vertical channel while the down velocity gathered on the basis of the fusion between altitude measurements from a barometric altimeter and an available vertical acceleration from a vertical accelerometer measurements every 10 Hz, and consequently the results of the estimation error in height and down velocity for the case of the multi- measurement mode-based LOAP and single mode EKF are almost the same with a slight difference between both of them being almost within less than 0.8 m in height and 0.7 m/s in down velocity as shown in Figure 16. These results clearly validate the advantages of the multi-measurement mode-based LOAP approach over the single mode EKF, especially when the observations from the GPS are not available during an interval of time. Consequently, the proposed technique guarantees the availability of the measurement data from various sensors in the absence of the information from the GPS receiver to keep describing the dynamics of the system.

## 6. Conclusions

In this paper, a multi-mode estimation technique-based measurement method for the small fixed wing UAV localization navigation problem is presented. The proposed approach depends on utilizing a Luenberger observer-based projection method as a continuous observer to estimate the navigation state. The projection consideration has been utilized to optimize the estimation performance. The simulation results prove that the Luenberger observer after projection (LOAP) is well suited to deal with this class of nonlinear systems. The positive symmetric definite matrix (P) and the observer gain matrix (L) are chosen simultaneously by solving a Lyapunov equation by means of a linear matrix inequality (LMI) algorithm. The positive symmetric definite matrix (P) is chosen as a solution of the Lyapunov inequality by means of the LMI technique to find the observer gain matrix (L). Convergence is achieved by utilizing LMI based on a Lyapunov stability inequality which keeps dynamic estimation error bounded by selecting the observer gain matrix (L). The state estimation is performed in switching environment between multiple active sensors to exploit the available information as much as possible and guarantee the availability of the measurement data, especially in GPS-denied environments. The proposed multi-measurement mode-based LOAP approach is implemented and compared with a single mode Extended Kalman Filter (EKF). The simulation results are divided into two parts based on the availability or not of the GPS signal. The results in the first part clearly show that the multi-measurement mode-based LOAP performs slightly better than single mode EKF, but we can say on the other hand that the EKF method gives better noise rejection for higher values of the measurement noise covariance matrix (*R*) while the proposed LOAP is well suited in the ideal case when the system not influenced by the noise. The results in the second simulation clearly show the superiority of the proposed multi-measurement mode-based LOAP over the single mode EKF, especially in absence of the GPS signal during an time interval because the proposed approach guarantees the availability of the position and velocity measurements in the absence of GPS and also the solution of the Lyapunov inequality-based stability to find the observer gain guarantees the stability of the dynamics estimation errors and keeps them bounded within an acceptable range. The drawback of the proposed approach is the high computation cost and need for high computational power-embedded systems, especially when dealing with high order state systems and that is because of the use of the computationally complex convex programming algorithm as a means to apply the LMI technique to solve the Lyapunov inequality-based stability to find the observer matrix gain (*L*). Therefore in general, if computational time requirements are not stringent and the number of states is less, then we can use the LOAP-based Lyapunov inequality stability while for a greater number of states, the familiar nonlinear Kalman filters perform quite well and faster.

## Figures and Tables

**Table 1:** The measured data and sampling rate used for each sensor.

Sensor	Measured Data	Frequency
GPS	Position and Velocity	1 Hz
Gyro	Angular Speed	10 Hz
Accelerometer	Acceleration	10 Hz
Barometric Altimeter	Height	10 Hz

**Table 2:** A comparison factors of the filters used in this study.

Observer	A Priori Statistical Information about Noises	Design of the Gain Matrix	Covariance Matrix (*P*)	Update Rate
LOAP	Not required (but introduced in the simulation for fair evaluation comparison)	Selected by solving the Lyapunov inequality by means of LMI techniques.	Selected as a solution of the Lyapunov inequality by means of LMI techniques.	10 Hz
EKF	Required	Using a correction matrix	A priori chosen, then estimated during the estimation process	10 Hz

**Table 3:** A standard deviations of estimated errors comparison between multi measurement modes based LOAP and single mode EKF.

Estimators	$\sigma_{\delta P_{n}}$ (m)	$\sigma_{\delta P_{e}}$ (m)	$\sigma_{\delta h}$ (m)	$\sigma_{\delta V_{n}}$ (m/s)	$\sigma_{\delta V_{e}}$ (m/s)	$\sigma_{\delta V_{d}}$ (m/s)
LOAP	2.2574	1.9289	0.5710	1.0717	1.1137	0.3703
SEKF	2.3330	1.9608	0.9666	1.0862	1.1314	0.4312
